# Role of the Ghrelin System in Colitis and Hepatitis as Risk Factors for Inflammatory-Related Cancers

**DOI:** 10.3390/ijms231911188

**Published:** 2022-09-23

**Authors:** Aldona Kasprzak, Agnieszka Adamek

**Affiliations:** 1 Department of Histology and Embryology, University of Medical Sciences, Święcicki Street 6, 60-781 Poznań, Poland; 2 Department of Infectious Diseases, Hepatology and Acquired Immunodeficiencies, University of Medical Sciences, Szwajcarska Street 3, 61-285 Poznań, Poland

**Keywords:** ghrelin system, inflammatory bowel diseases, hepatitis, liver fibrosis, inflammatory-related cancers, colorectal carcinoma, hepatocellular carcinoma

## Abstract

It is not known exactly what leads to the development of colorectal cancer (CRC) and hepatocellular carcinoma (HCC), but there are specific risk factors that increase the probability of their occurrence. The unclear pathogenesis, too-late diagnosis, poor prognosis as a result of high recurrence and metastasis rates, and repeatedly ineffective therapy of both cancers continue to challenge both basic science and practical medicine. The ghrelin system, which is comprised of ghrelin and alternative peptides (e.g., obestatin), growth hormone secretagogue receptors (GHS-Rs), and ghrelin-O-acyl-transferase (GOAT), plays an important role in the physiology and pathology of the gastrointestinal (GI) tract. It promotes various physiological effects, including energy metabolism and amelioration of inflammation. The ghrelin system plays a role in the pathogenesis of inflammatory bowel diseases (IBDs), which are well known risk factors for the development of CRC, as well as inflammatory liver diseases which can trigger the development of HCC. Colitis-associated cancer serves as a prototype of inflammation-associated cancers. Little is known about the role of the ghrelin system in the mechanisms of transformation of chronic inflammation to low- and high-grade dysplasia, and, finally, to CRC. HCC is also associated with chronic inflammation and fibrosis arising from different etiologies, including alcoholic and nonalcoholic fatty liver diseases (NAFLD), and/or hepatitis B (HBV) and hepatitis C virus (HCV) infections. However, the exact role of ghrelin in the progression of the chronic inflammatory lesions into HCC is still unknown. The aim of this review is to summarize findings on the role of the ghrelin system in inflammatory bowel and liver diseases in order to better understand the impact of this system on the development of inflammatory-related cancers, namely CRC and HCC.

## 1. Introduction

Colorectal cancer (CRC) is one of the most common human malignancies worldwide, being third in terms of incidence and second in terms of mortality in 2018 [1,2,3]. This histologically heterogeneous group of cancers includes mostly classic adenocarcinomas (90–95% of all cases), but also rare histotypes which are often under-recognized [4,5].

Liver cancer is predicted to be the sixth most commonly diagnosed cancer and the fourth leading cause of cancer death worldwide in 2018 [1]. Histologically, hepatocellular carcinoma (HCC) constitutes 70–85% of the primary liver cancers. HCC occurs in ~85% of patients diagnosed with cirrhosis. Most cases of this cancer (~80%) are related to hepatitis C (HCV) or hepatitis B virus (HBV) infections [1,6]. Similarly to viral liver diseases that cause cirrhosis and HCC, nonalcoholic fatty liver disease (NAFLD) also increases the risk of liver cancer [7]. NAFLD can progress to nonalcoholic steatohepatitis (NASH), where inflammation plays a central role in the liver’s response to injury and contributes to its chronicization. However, the mechanisms of NAFLD progression to NASH are still not clear [8,9,10,11].

Chronic inflammation increases the risk of many cancers, including CRC [12,13,14] and HCC [15]. HCC is growing quickly as a result of immunosuppression and reprogramming of the metabolism [16]. In both CRC [17] and HCC [18], stromal and immune cells present in the tumor microenvironment (TME), and/or in the circulation, may play a role in immunosuppression, immune-mediated tumor progression, and tumor metastases. However, the molecular mechanisms of these processes and their clinical consequences are poorly understood.

The ghrelin system, which plays an important role in physiology and pathology of the gastrointestinal (GI) tract [19,20,21,22], includes a complex family of peptides, namely active (acylated) ghrelin (AG) and a growing number of alternative peptides (e.g., unacylated ghrelin (UnAG), obestatin, In1-, and In2-ghrelin), known growth hormone secretagogue receptors (GHS-Rs), and unknown receptors, as well as modifying enzymes (e.g., ghrelin-O-acyltransferase (GOAT) [19,21,23,24,25,26,27].

The orexigenic effect of ghrelin is most commonly described, controlling food intake, energy expenditure, adiposity, and growth hormone (GH) secretion. It promotes various physiological effects, including energy metabolism and amelioration of inflammation. The role of ghrelin signaling as an important mechanism regulating immunometabolism and inflammatory aging is also highlighted [28]. A number of papers are emerging comparing the effects of ghrelin and obestatin as “sibling proteins” encoded by the same gene, including their involvement in inflammation and colon carcinogenesis [29].

The ghrelin system plays a role in the pathogenesis of inflammatory bowel diseases (IBD), which are well known risk factors for the development of CRC [30,31,32,33] and inflammatory liver diseases, which can trigger the development of HCC [9,10,34,35,36,37].

A number of mechanisms have been described for the protective effects of ghrelin on the GI tract, e.g., reducing the inflammatory response, decreasing oxidative stress in mucosa, improving intestinal barrier function, increasing cellular vitality and proliferation, and restoring normal microcirculation [22,38,39]. Little is known about the role of ghrelin system components in the mechanisms of transformation of chronic inflammation to low- and high-grade dysplasia (LGD, HGD), and, finally, CRC.

Data on the effects of the ghrelin system on hepatic lipid metabolism (including the development of NAFLD) are controversial. Some in vitro studies indicate that AG enhances lipogenesis (and NAFLD), activating GHS-R on hepatocytes and leading to an increase in triglyceride (TG) synthesis [40], while other studies indicate a decrease in TG accumulation with concomitant increases in glutathione peroxidase (GPx) in high-fat diet (HFD)-induced NAFLD and AG treatment [41]. In animal and in vitro models of liver injury, the anti-inflammatory and antioxidant effects of AG in the liver have been described, linked to a reduction in fibrosis [34,41,42].

The aim of this review is to summarize findings on the role of the ghrelin system in inflammatory bowel and liver diseases in order to better understand the impact of this system on the development of inflammatory-related cancers, namely CRC and HCC.

## 2. The Possible Mechanisms of Cancer-Related Inflammation in Colon and Liver

The cancer-related inflammation (CRI) was recognized as the seventh characteristic feature of cancer [43] and added to the other six [44]. Chronic inflammation in the colon, as well as increased turnover of epithelial cells, appear to play a role in the development of LGD and HGD, which may further convert into CRC [45]. It is clear that some of the important molecular mechanisms in the development and progression of CRC are signaling pathways based on inflammatory mediators in mucosa (e.g., cyclooxygenase 2 (COX-2), interleukin 6 (IL-6), IL-23, tumor necrosis factor α (TNF-α), nuclear factor κB (NF-κB), and chemokines) (reviewed in: [46]). Moreover, the presence of specific immune cells in TME plays a major role in the progression of CRC and HCC, whose products enhance proliferation and survival of malignant cells, as well as affecting angiogenesis and tumor metastasis and reducing response to treatment [17,18,47]. Abundant evidence suggests that TME significantly affects the phenotypes of GI tract tumors (including CRC and HCC). In the case of CRC, TME connects the immunological phenotypes of primary and metastatic tumors, helping to identify pro-metastasis components [47].

In HCC, the impact of different types of microenvironments on primary liver cancer (PLC) phenotypes generated by distinct oncogenes is still unclear (reviewed in: [48]). CRI-associated mediators can induce genetic instability, leading to the accumulation of random genetic changes in tumor cells [15,17,43,49,50]. A list of biological properties that are key to modifying the TME and educating malignant, stromal, and inflammatory cells toward the metastatic phenotype of CRC is provided. These include the plasticity of immune cells in response to paracrine and autocrine signals, as well as the acquisition of immune-like phenotypic features (NT5E/CD73^+^, CD68^+^ and CD163^+^) by tumor cells, which increase interactions with TME components through the production of immunosuppressive mediators [17].

All types of non-specific inflammatory bowel diseases (IBDs), namely ulcerative colitis (UC), Crohn’s disease (CD), and microscopic colitis, are risk factors for the development of CRC [45,46,51,52]. The relationship between IBDs and CRC has been known about for more than 100 years, when Antoni Leśniowski (1904) and Burill Bernard Crohn et. al. (1925) published their findings in native journals [45]. Epidemiological data indicate that patients with IBD have a 1.7-fold increased risk of CRC [53]. In CRC, the colitis is often considered the initial trigger for CRI [14]. IBD-related CRC accounts for about 2% of overall annual CRC mortality, but for 10–15% of annual deaths in patients with IBD [45]. Patients with IBD-related CRC tend to be younger than those with sporadic CRC, and have a 5-year survival rate of 50%. Long-term prognosis of CRC may be poorer in patients with IBD than in those with sporadic CRC [45,54,55]. A recent meta-analysis found a significant family prevalence of CRC among patients with IBD. American countries and first-degree relatives were found to have a higher prevalence of both disease processes [56]. In a recent meta-analysis on risk factors for advanced colorectal neoplasia, HGD, or CRC in IBD patients, histologic inflammation was considered a risk factor in the multivariate analysis (OR/HR > 1), but there was weak evidence for histologic inflammation [57]. Considering the inflammatory factor itself, the risk of CRC in IBDs is increased by disease activity, severity of inflammation, and postinflammatory polyps. IBD-associated CRC is often characterized by multiple neoplastic lesions, with a higher proportion of mucinous and signet-ring cell carcinomas contributing to its aggressiveness [45,46].

Most cases of HCC arise from chronic inflammation associated with HCV/HBV infections and within a fibrotic liver [15,58]. Other etiological factors include alcohol abuse, NASH associated with metabolic syndrome (MetS) or type 2 diabetes mellitus (T2DM), autoimmune liver disease, drug-induced liver injury, and aflatoxin exposure [8,58]. Liver cirrhosis, which coexists in many patients with HCC, is associated with local and systemic immune deficiency [15]. Disruption of the liver’s immune network, which provides systemic protection while maintaining immunotolerance, is a hallmark of chronic liver disease and HCC [59]. In oncogenic initiation, HBV and HCV can induce immune reactions leading to progressive liver cell damage that ultimately leads to the development of HCC [60]. Inflammation in the liver can, therefore, result in oncogenesis and immune cell dysfunction (reviewed in: [15]). The immune cells in TME play a variety of critical roles in the development and progression of HCC. The close contact and interaction between tumor-infiltrating T cells and B cells, increasing local immune activation, improves the prognosis of patients with HCC [61].

In viral-associated HCC (especially those of long duration and high viral load), an ineffective antiviral host immune response causes immune-associated damage. Repeated hepatitis caused by the inflammation-necrosis-proliferation cycle leads to the production of reactive oxygen species (ROS) that promote genetic mutations, fibrosis, cirrhosis, and HCC [62]. In the case of HCV infection, the main causative factors of HCC include an increase in oxidative stress along with hepatic steatosis induced by the HCV core protein, in addition to changes in cellular gene expression, e.g., TNF-α, and alterations in intracellular signaling pathways, including c-Jun N-terminal kinase (JNK). In the case of chronic HBV infection, the carcinogen is primarily the HBx protein, affecting the proliferation and apoptosis of liver cells. HBx acts similarly to oncogenes such as c-Myc or adenoviral E1A protein. It should be mentioned that in addition to typical viral infections, an increase in non-A non-B hepatitis contributes to the development of HCC [49,62].

## 3. The Ghrelin System—General Overview

**Figure 1:**
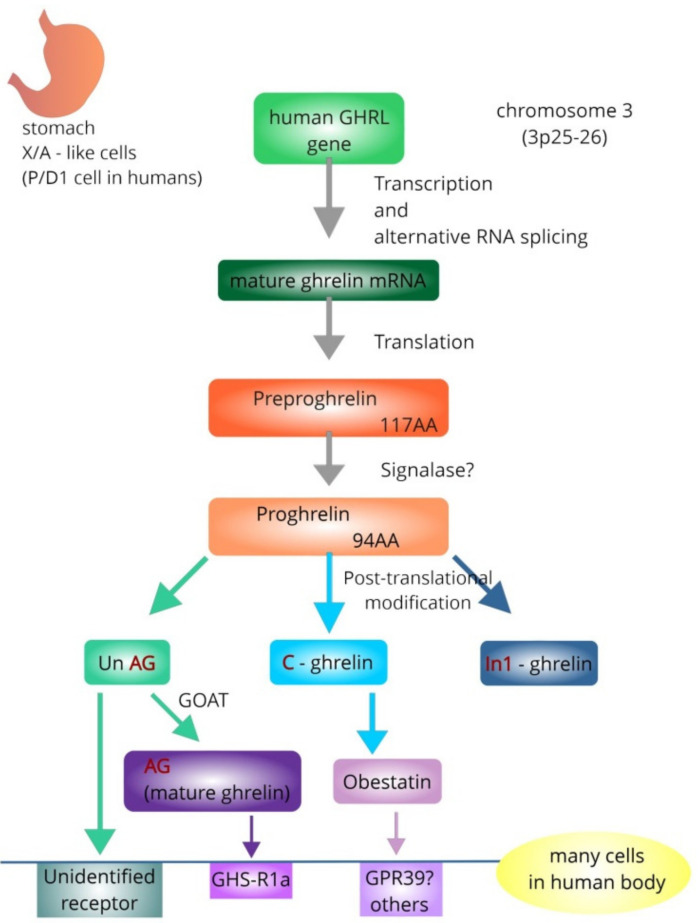
Simplified representation showing formation of mature, acylated ghrelin (AG) and obestatin from human ghrelin gene (*GHRL*). The *GHRL* contains six exons and four introns in total, and the introns enable alternative splicing [66]. The AG strongly binds growth hormone secretagogue receptor 1a (GHS-R1a), which is responsible for its GH-releasing capacity and the majority of its biological functions [21,63,73,74]. The proposed receptor for obestatin is GPR39, but other receptors are not excluded. The receptor for unacylated ghrelin (UnAG) is undetermined. [AA—amino acids; GOAT—ghrelin-O-acyltransferase; GPR39—the orphan G protein-coupled receptor].

The main product of the ghrelin gene (*GHRL*), located on the short arm of chromosome 3, is a 28-amino acid (AA) peptide called ghrelin, which is a natural endogenous ligand for pituitary GHS-R, and a potent stimulator of GH release. The native (canonical) ghrelin, occurring in acylated (active ghrelin or ghrelin), and unacylated (other terms: inactive, des-acylated ghrelin) forms (AG and UnAG, respectively), was the first peptide identified among the products of the *GHRL* [63,64,65,66] (Figure 1). Obestatin, a 23-AA peptide derived from the carboxy-terminal portion of the ghrelin precursor, was isolated from the stomachs of rats by Zhang et al. The stomach seems to be a major source of circulating obestatin [67].

The human GHSR gene is composed of two exons, whose alternative splicing can form two mRNAs, named GHS-R1a and GHS-R1b [68,69]. The first transcript, which includes exons 1 and 2, encodes a 366-AA G protein-coupled receptor (GPCR) with seven transmembrane domains (TMDs). GHS-R1b mRNA generates a truncated 289-AA GPCR isoform with only five TMDs, whose role is still unclear. GHS-1Ra strongly binds only ghrelin modified with Ser3 acylation, i.e., AG [70]. It is interesting to point out that recent studies show that the acetylation process itself takes place in the liver [71]. The process is catalyzed by GOAT, a membrane-bound enzyme that attaches eight-carbon octanoate to a serine residue in ghrelin and thereby acylates inactive to produce active ghrelin [21,72]. Thus, the active form of ghrelin has the ability to bind GHS-R1a, and is responsible for its GH-releasing capacity and the majority of its biological functions [21,63,73,74].

The circulating ghrelin is secreted mostly by endocrine cells (X/A-in rats and P/D1 in humans) of the oxyntic (parietal) mucosa of the gastric fundus [20,75]. Ghrelin is secreted periodically, with a decrease in its secretion in the morning. It increases before meals and decreases after meals [63,76]. Chronic caloric restriction (CR) during aging increases plasma ghrelin concentrations as well as total ghrelin production in the stomach of rodents, and reverses age-related loss of GHS-R expression in pituitary [77]. Calorie restriction exerts an extraordinary anti-inflammatory effect in humans as well. The increase in ghrelin production reflects one of the anti-inflammatory effects initiated by CR [78]. Hence, it is also hypothesized that reduced levels of ghrelin may be responsible for the “anorexia of aging”, an increase in chronic low-grade inflammation, a decreased T-cell response, and reduced muscle and bone mass due to reduced GH levels in humans [79].

Ghrelin acts in the so-called secondary peripheral circadian clock (or non-suprachiasmatic nucleus) along with other hormones (e.g., melatonin, GH, insulin, and adiponectin), playing an important role in maintaining circadian rhythms in the brain and peripheral organs [80]. It is worth noting that circulating ghrelin can be rapidly desoctanoylated by plasma esterases such as butyrylcholinesterase, forming an UnAG [81]. It is important to remember that ghrelin circulates in the blood mainly under its inactive form [82,83]. The UnAG can be re-acylated in vivo via long chain fatty acids and GOAT. It plays an important role in metabolism and food intake, probably by balancing the circulating AG [83]. The physiological role of UnAG appears to be antagonistic to AG [24]. AG is a major appetite modulator at the central nervous system level that induces many tissue-specific metabolic effects, whereas UnAG appears to be an independent hormone that directly reduces ROS formation in skeletal muscle, also by increasing autophagy, with associated improved tissue inflammation and insulin activity [20]. Ghrelin also reduces the process of autophagy in several inflammatory conditions (e.g., acute hepatitis, liver fibrosis, or obesity-associated adipose tissue inflammation) to prevent further cell injury [84,85,86,87].

Secretion of obestatin, like ghrelin, is also pulsatile and displays an ultradian rhythmicity [88]. In contrast to the effects of ghrelin, treatment of rats with obestatin inhibited food intake, suppressed intestinal contraction, and reduced weight gain [67]. Although obestatin seemed to exhibit actions opposite to ghrelin, it is “a multi-functional peptide hormone in its own right” [89]. The discoverers of this hormone considered the receptor for obestatin as the orphan GPCR called GPR39 [67], although this has proven to be debatable and not confirmed by other authors [90]. GPR39 belongs to a large family of 7-TM containing GPCR, which can be activated by changes in extracellular Zn^2+^ in physiological concentrations. It is now thought that some of the effects of obestatin may indeed have been mediated by GPR39, although the research continues for ligands other than Zn^2+^ for GPR29 [91].

Tissue expression of ghrelin in animals and humans was identified in most central and peripheral tissues [92,93,94,95]. GHS-R1a expression was also widespread in all human tissue studied [92]. Notably, mRNA expression of ghrelin, both GHS-Rs (GHS-R1a and GHS-R1b), and GOAT is also detected in the normal colon and human liver [71,92,96,97,98,99]. GHS-R transcript expression was detected in human hepatocytes and activated hepatic stellate cells (HSCs), but not in quiescent HSCs [42]. GHS-R1a (mRNA, protein) was also detected in the liver and hypothalamus derived from wild-type (WT) mice, whereas *ghsr1a^(-/-)^* mice showed no expression [40]. Similarly, GHS-R1a was detected in Kupffer cells isolated from WT mice but not in GHS-R1a deficient animals [36].

Obestatin expression is demonstrated in a wide range of animal and human tissues and, in the case of the GI tract, also from the cardia to the ileum and in the pancreas [93,94,100]. Although obestatin was also detected in the rat colon [94], this peptide was not detected in the human colon, rectum, or liver [93].

Both ghrelin and GHS-Rs were also detected in the lymphoid system organs (human spleen, lymph nodes, and thymus) [92,101]. By contrast, there was no such immunoreactivity to obestatin in human lymphoid organs (tonsil, thymus, appendix, spleen) [93]. The cells from the lymphatic system with mRNA expression for ghrelin and GHS-R1a include, such as B and T cells, neutrophils, macrophages, monocytes, dendritic cells and NK cells. Among the cells of the lymphatic system with mRNA expression for ghrelin and GHS-R1a are: B and T cells, neutrophils, macrophages, monocytes, dendritic cells, and NK cells [101,102,103,104]. In vivo studies in a mouse model as well as in vitro studies have shown the existence of a reciprocal regulatory network through which ghrelin and leptin control immune cell activation and inflammation. They indicate that ghrelin and GHS-R are expressed in human T lymphocytes and monocytes, where ghrelin acts through GHS-R to specifically inhibit the expression of proinflammatory cytokines (e.g., IL-1β, IL-6 and TNF-α). Ghrelin leads to an inhibition of leptin-induced cytokine expression. In addition, ghrelin exerts potent anti-inflammatory effects and alleviates endotoxin-induced anorexia in a mouse model of endotoxemia [101]. Waseem et al. showed the expression of ghrelin and GHS-Rs in mouse macrophages. The inhibitory effect of exogenous ghrelin (eG) on the production of proinflammatory cytokines (IL-1β and TNF-α) and the increased release of anti-inflammatory cytokines (IL-10) in lipopolysaccharide (LPS)-stimulated macrophages was demonstrated. The putative mechanisms of action of ghrelin on the production of these inflammatory mediators have been reported, most notably decreasing NF-κB activation and increasing mitogen-activated protein kinase (MAPK) activation [103]. The presence of GHS-R1b was also confirmed in immune tissues, but it was not activated by ghrelin [105]. While the participation of the ghrelin system in immune responses mostly concerns T cell response (e.g., Th1, Th17, and Tregs), it is also implicated in innate immunity (toll-like receptors, TLRs). The therapeutic effect of ghrelin is also mediated by the release of other anti-inflammatory factors, e.g., endogenous GH and insulin-like growth factor 1 (IGF-1) [106,107]. In an animal model, ghrelin was also shown to attenuate mechanical hyperalgesia, reduced spinal cord TNF-α and IL-1β levels. Thus, it can be involved in mechanisms of neuropathic pain [108].

The effects of different factors on ghrelin levels under conditions of acute inflammation have also been demonstrated. One mechanism for the inflammation-induced downregulation of ghrelin (similar as LPS) may be the effect of IL-1β on gastric mucosa cells, which in turn produce prostacyclin as a second messenger [109].

The role of obestatin in both GI tract physiology and pathology is emphasized (reviewed in: [29,110]). With regard to the objectives of the current work, the role of obestatin will be highlighted mainly in the interaction in the inflammatory process associated with CRC and HCC.

## 4. The Effects of Ghrelin System on the Intestinal Inflammation

Intestinal inflammation is a risk factor for GI tract cancers, with the ghrelin system playing a pathophysiological role in regulation of a range of immunological functions through the GHS-R [31,51,111,112]. Multiple effects of ghrelin as a potential anti-inflammatory mediator and its potential therapeutic use in inflammatory diseases and injury are summarized in the following reviews [104,113,114]. In contrast to the large number of studies on the anti-inflammatory effects of ghrelin, research on obestatin in this area is limited [115,116,117,118]. The therapeutic effect of obestatin was associated (like ghrelin) with inhibition of inflammation and activation of anti-inflammatory cytokines [115]. The mechanisms of obestatin’s anti-inflammatory effects are not always clear, and the presence of multiple signaling pathways leading to such effects has been highlighted [110]. The involvement of components of the ghrelin system in types of colitis other than typical IBD should also be highlighted, e.g., infectious, ischemic, and drug-induced colitis (reviewed in [119]).

### 4.1. Clinical Studies

The ghrelin system demonstrates mainly anti-inflammatory properties, through the regulation of both anti-inflammatory (e.g., IL-10, IL-4, transforming growth factor β (TGF-β) (stimulation)), and proinflammatory cytokines (e.g., TNF-α, interferon gamma (IFN-γ), IL-1β, IL-6 (inhibition)). It can also play a role in the pathogenesis of human IBD [31,33,112,120,121], including UC, CD, and microscopic colitis [51]. Active intestinal inflammatory process increases endogenous ghrelin production [120]. The IBDs also penetrate the visceral adipose tissue (AT), which may be an additional source of cytokines and adipokines (including ghrelin) responsible for the inflammatory process in these diseases (reviewed in: [33]).

Serum ghrelin concentrations were demonstrated to be increased in IBDs compared to healthy individuals, and higher in active forms of UC and CD compared to patients in remission [122,123,124,125,126,127,128,129]. Positive correlations were described between AG levels and erythrocyte sedimentation rate, fibrinogen, and C-reactive protein (CRP), and negative with IGF-1 and nutritional status parameters (e.g., BMI, fat mass, fat free mass) [123,124]. Moreover, positive correlations were demonstrated between ghrelin and TNF-α levels in active IBD [123]. Some studies indicate significantly higher ghrelin concentrations depending on the location of inflammatory lesions in CD, e.g., in the ileum (higher) compared to the colon (lower) [122]. Only one study found reduced levels of ghrelin in IBD cases (CD and UC) compared to their healthy counterparts (both non-fasting and fasting) [129], and only one study which does not confirm the changes in ghrelin levels in IBDs vs. the control group [130]. In order to clarify the relationship between ghrelin and IBDs, it is suggested that two forms of ghrelin (AG, UnAG) should be tested for more reliable results [30]. Standardization of sample preparation is needed to ensure reliable measurements of ghrelin concentrations [131,132].

Regarding serum obestatin levels as a biological marker of inflammatory activity in IBD patients, there are only few studies [125,128]. In contrast to ghrelin, only borderline higher mean obestatin values (217.4 ± 59.8 pg/mL) in active vs. inactive disease (189.0 ± 46.8 pg/mL (*p* = 0.0607) or no significant differences in serum levels of this biomarker between such patient groups have been shown [128]. Interestingly, a significantly lower obestatin/ghrelin concentration ratio was found in active IBD than in patients in remission [125,128]. A statistically significant correlation was also found between the obestatin/ghrelin ratio and disease activity [125]. The authors conclude that ghrelin levels or the obestatin/ghrelin ratio may serve as markers of inflammatory activity in UC patients [128].

Studies on tissue expression of the ghrelin system in IBDs in vivo also indicate increased ghrelin production in the colon mucosa of active UC patients compared to patients in remission [128], as well as increased GHS-R1a mRNA in active CD compared to the control group [39,121]. No such differences were found in the expression of colonic mRNA of obestatin in active disease vs. UC in remission [128].

In conclusion, clinical studies indicate that serum concentrations of ghrelin (rather than obestatin) can be used as valuable markers of disease activity and the degree of mucosal damage in IBD. They can be used together with other markers of inflammatory activity in IBD, e.g., CRP level [127].

The role of ghrelin system components in CRC itself was described in an earlier review [133]. Studies showing reduced ghrelin concentrations in CRC suggest a role for ghrelin in creating a metabolic proinflammatory environment in the early stages of CRC development, resulting in enhanced tumor growth. However, a scenario is also assumed in which reduced serum ghrelin levels in CRC patients are secondary, as a result of the inhibitory effect of other tumor factors/hormones on its production with tumor progression [134].

### 4.2. Animal Models of Colon Inflammation

In order to better understand the role and mechanisms of intestinal inflammation in IBD-associated colorectal cancer, experimental models of colitis in animals (rats, mice) are used. Animal models allow for the understanding of the mechanisms of mucosal immunity, the definition of the intestinal microbiome under different conditions, and thus the understanding of the influence of intestinal microorganisms on inflammation. Mice with specific genetic and/or immunological defects are also being studied (reviewed in: [135]).

Among the most commonly used models of colitis, and thus the study of the pathogenesis of human IBD, are the following: dextran sodium sulphate (DSS)-, 2,4,6-trinitrobenzene sulfonic acid (TNBS)-, and azoxymethane (AOM)/DSS-induced colitis [135,136,137]. The hallmark of the TNBS-colitis model is the development of transmural inflammation, which closely resembles the histopathological changes that occur in human CD [136]. DSS-colitis causes human ulcerative colitis-like pathologies due to its toxicity to colonic epithelial cells, resulting in a loss of epithelial barrier function and the entry of organisms residing in the intestinal lumen or their products into the lamina propria. This results in the stimulation of innate and adaptive lymphoid elements and the secretion of proinflammatory cytokines and chemokines. There is an influx of cells with cytotoxic potential, such as neutrophils and macrophages [135]. A less commonly studied mouse model of IBD is *Citrobacter rodentium* infection, which also leads to colitis. One of the clinicopathological features is diarrhea and colon hyperplasia. However, the key mechanisms for successful elimination of the *C. rodentium* infection appeared to be upregulation of Th17 and Treg pathways in distal colon as well as increased expression of ghrelin compared to uninfected mice [138].

In the context of better understanding colitis-associated CRC, the most commonly used animal model is the AOM/DSS colitis model [12,137], as this model closely mimics the histological, pathological, and molecular features of colitis-associated cancer in humans [137]. AOM/DSS model effectors include factors such as: inflammatory cells (e.g., macrophages, lymphocytes, and plasma cells), NF-κB, Janus kinase/signal transducer and activator of transcription 3 (JAK/STAT3) pathway, proinflammatory cytokines, and β-catenin (reviewed in: [137]). As has been confirmed by the authors of a recent review, among the many proinflammatory cytokines, TNF-α and IL-1β are key in the development of CRC [13].

In DSS- and TNBS-induced colitis, an increase in production of ghrelin and/or GHS-Rs mRNA was demonstrated, as compared to the control [39,139,140]. The protective effect of ghrelin, as well as that which accelerates the healing of damaged colon mucosa, results from an increase in COX-2-derived prostaglandin E2 (PGE2) and improved sensory nerve integrity, with an increase in neuropeptides from sensory afferent endings (e.g., calcitonin gene-related peptide, CGRP) [39]. Recent studies on the *ghsr^(^^−/−)^* mouse model have shown that suppression of GHS-R increases intestinal inflammation during aging and increases vulnerability to colitis [141].

Overall, the main anti-inflammatory effects of the ghrelin included: lowered inflammation (especially in chronic colitis) on both the tissue and the systemic level, as well as disease relapse prevention [38,115], healing of colonic lesions in mucosa with spontaneous regeneration of the colon [39,107,142,143,144,145,146], maintenance of intestinal barrier function [143], improved blood flow and increased cell proliferation in mucosa [107,145], increased DNA synthesis in the colon mucosa [145], and protection of the intestinal mucosa from sepsis-related injury [32]. The cytoprotective role of ghrelin in colitis also appears to depend on the dose of eG administered. Only a moderate to high dose of the peptide showed beneficial effects (mediated by GHS-R1a) on colitis by suppressing inhibitory κB-α (IκB-α) degradation and reducing nuclear expression of NF-κB p65. This resulted in myosin light chain kinase (MLCK) inhibition and phosphorylated myosin light chain 2 (pMLC) activation, preventing the loss of tight junctions [143].

In turn, in mouse models of acute TNBS- [139] and DSS-induced colitis [72,140,147], attention was brought to the proinflammatory effects of ghrelin. An increase in ghrelin and GHS-R mRNA expression was demonstrated in TNBS-induced colitis in mice. However, the effect of eG on inflammatory markers in colonic mucosa was not studied in this work [139]. Other studies confirm the upregulation of the inflammatory process by endogenous ghrelin. Furthermore, administration of eG enhanced the clinical disease activity and promoted infiltration of neutrophils and colonic IL-1β levels. Decreased myeloperoxidase (MPO) activity and IL-1β levels were only observed in ghrelin knockdown mice models [147].

The role of GHS-Rs in colitis pathogenesis in a mouse model of DSS-induced colitis was also clarified. The authors observed lower colonic macrophage infiltration and TLR expression in *ghsr^(−/−)^* compared with WT mice [140]. Interesting observations were made by Tian et al. in DSS-induced colitis and knockdown of GOAT (*GOAT*^(*−/−)*^) mice. The authors reported a decrease in colitis-induced inflammation responses and apoptosis in *GOAT^(−/−)^* mice. In contrast, GOAT overexpression significantly exacerbated colitis, which could indicate a proinflammatory function of GOAT [72]. Zhang et al. demonstrated an anti-apoptotic effect of ghrelin via GHS-R1a in both mouse models of IBD. Furthermore, ghrelin modulated the unfolded protein response (UPR) pathway and inhibited cell apoptosis [146].

Interesting studies on the administration of exogenous AG in the absence of endogenous ghrelin (*Ghrl* deletion) were performed in two mouse models of colon carcinogenesis. These were genetic (*Apc^(^^Min/+)^* mice) and inflammation-associated (AOM/DSS treatment) models. In inflammation-induced colitis, administration of eG significantly inhibited tumor formation in the colon. In contrast, ghrelin administration had no effect on the number of intestinal tumors forming in *Apc^(^^Min/+)^* mice. While the absence of endogenous ghrelin did not affect the incidence of intestinal tumors in either AOM/DSS treated and *Apc^(Min/+)^* mice, the size of the tumors was larger in the *Ghrl^(−/−)^* colon. Interestingly, no tumor-promoting effect was observed after eG administration in any of the models [148].

In an animal model of colitis, obestatin, similarly to ghrelin, was shown to significantly improve the clinical and histopathological features of chronic colitis, while being less effective in the acute colitis. As with ghrelin, obestatin’s anti-inflammatory effects include the inhibition of NF-κB, TNF-α, IL-1β, IFN-γ, and IL-6, and increasing levels of anti-inflammatory cytokines (IL-10 and TGF-β) [115,116,117,118]. Obestatin attenuated histological damage in colitis by reducing the polymorphonuclear leukocyte infiltration (as evaluated by tissue MPO levels), inhibiting production of ROS and proinflammatory Th1 cytokines in both acute and chronic colitis, and stimulating the synthesis of anti-inflammatory cytokines in chronic colitis [115,117]. Other studies have confirmed a dose-dependent, protective effect of obestatin on damaged colonic mucosa. A reduction in the area of TNBS- and acetic acid-induced colitis and an increase in mucosal cell proliferation were observed. This effect was associated with improved mucosal blood flow in the colon and reduced local and systemic inflammatory processes [117,118]. For the most part, ghrelin and obestatin have complementary effects on colon inflammation [29]. The relationship between obestatin in benign and malignant lesions of the organs of interest (colon, rectum) is less understood.

### 4.3. In Vitro Models of Colon Inflammation

Various in vitro models of colitis have demonstrated that ghrelin is responsible for modulating the responses of effector T cells (Th cells) by affecting their proliferation (inhibits) and apoptosis (induces). It has been observed that the absence of ghrelin signaling in Th cells resulted in a significant worsening of colitis with increased inflammation dependent on pathological accumulation of CD4 effector T cells in the lamina propria [149]. The cytoprotective effect of ghrelin on damaged colonic mucosa was shown to occur due to the antioxidant activity of this peptide. The formation of malondialdehyde (MDA), the end product of lipid peroxidation, is the main indicator of oxidative damage. Similarly to animal models studies, a decrease in ROS via the activity of catalase (CAT) and manganese superoxide dysmutase (MnSOD) was also reported in human colon HCT116 cells after administration of eG [150].

In turn, GHS-Rs appear to play a role in direct and indirect anti-inflammatory [121,143] and anti-apoptotic [146], but also proinflammatory, activities [139,140,151]. Significantly fewer proinflammatory cytokines were found in LPS-stimulated macrophages in vitro derived from *ghsr^(−/−)^* mice compared to WT mice. In turn, administration of a GHS-R antagonist reduced proinflammatory cytokines in LPS-stimulated WT mouse-derived macrophages [140]. Proinflammatory effects of ghrelin are also suggested by studies in non-transformed human colonic epithelial NCM460 cells transfected with a functional GHS-R. The authors suggest that ghrelin could participate in colitis pathophysiology through the induction of protein kinase C (PKC)-dependent NF-κB activation, and TNF-α-induced IL-8 gene expression at colonocyte levels [139].

Furthermore, ghrelin has been shown to be involved in up-regulation of COX-2 protein levels and its promoter activity, leading to a significant increase in PGE2 secretion. In addition, stimulation of the cAMP responsive element-binding protein (CREB) phosphorylation has been demonstrated, mainly via PKCδ activation and direct stimulation of PKCδ phosphorylation via ghrelin [151]. The anti-apoptotic effect of ghrelin was confirmed in human Caco-2 cells induced by TNF-α [146].

There is a lack of in vitro studies on the effect of obestatin in the colon inflammation.

The immunomodulatory role of the ghrelin system, as confirmed by several studies performed on animal models and in vitro studies, is presented in Table 1. Most of these colitis models confirm the protective effect of the ghrelin system on the inflammation-damaged intestinal mucosa, which may have clinical implications (reducing inflammation, indirect anti-cancer effects, or an additional form of therapy for these conditions). However, most of these studies have not investigated the exact mechanisms of the ghrelin system in colon carcinogenesis.

Table 2 provides a short summary of the potential role of ghrelin and obestatin in IBD colitis.

## 5. The Effects of Ghrelin System on the Hepatic Inflammation

### 5.1. Clinical Studies

Epidemiological data show parallel increases in obesity, T2DM, NAFLD/NASH, and HCC [7,8,152]. NAFLD is now the fastest growing cause of HCC in the USA, France, and the UK [7], but the pathogenesis of this condition is still unclear [9]. Inflammation, oxidative stress, and apoptosis as cellular processes play a critical role in the progression of NAFLD/NASH [10]. The excellent reviews present risk factors and the potential involvement of the ghrelin system in the mechanisms of NAFLD initiation and progression [10,11].

In NASH, which is a high risk factor for fibrosis and HCC, differential serum AG and UnAG levels in adults and children were observed. Lower serum AG levels have been demonstrated in NASH patients compared to controls [153,154], and UNAG levels were twice as high in morbidly obese NASH vs. non-NASH patients [155]. Moreover, in NASH, with more advanced fibrosis, UnAG concentrations were almost twice as high as in patients with less liver fibrosis [155]. Another study including morbidly obese individuals with NAFLD (including 13% with NASH) showed a correlation between ghrelin levels and diabetes [156], whereas, in obese prepubertal children, a significant correlation between ghrelin concentrations and elevated immunoglobulin levels and liver function index was observed [157].

The results of studies on ghrelin concentrations in the whole group of chronic liver diseases (CLD) of various etiologies (e.g., virus hepatitis, biliary/autoimmune, alcohol/cryptogenic, and others), are also highly variable. Significantly elevated ghrelin levels have been observed in all CLD patients compared to healthy volunteers. Serum ghrelin levels were elevated in Child class C liver cirrhosis compared to CLD with no cirrhosis. However, in these studies, ghrelin levels did not correlate with liver function, but with some clinical complication signs (e.g., ascites and encephalopathy) and biochemical parameters (e.g., inflammatory markers). In the HCC group, a strong inverse correlation between α-fetoprotein (AFP) and ghrelin levels was observed. Interestingly, no significant differences were found for ghrelin serum levels comparing different etiologies of CLD [158]. In another study, ghrelin levels were lower in both patients with alcoholic hepatitis and chronic hepatitis C (CHC) compared with control. Moreover, ghrelin concentration was lower in patients with advanced fibrosis (Metavir score 3–4) than in those with mild fibrosis (Metavir score 0–2) [42]. A study in patients with HCV-associated liver cirrhosis from Egypt showed that plasma ghrelin is a good marker of malnourishment [159]. Another study in pediatric cirrhotic patients showed that decreased AG levels and increased UnAG levels were associated with cirrhosis severity [160]. More recent studies indicate lower levels of ghrelin in cirrhotic patients (both compensated and decompensated) as compared with normal subjects. According to the authors, this peptide can be used as a serum marker for detection and assessment of the severity of liver cirrhosis [161]. In contrast, in alcoholic cirrhosis, AG levels have been shown to be elevated vs. controls, with apparent preservation of normal postprandial gastric ghrelin secretion mechanisms. GH levels were also elevated, with no correlation with AG in cirrhotic patients, but confirmed in healthy subjects. Despite increased secretion of ghrelin and GH, patients with alcoholic cirrhosis remain anorexic and catabolic, suggesting potential tissue resistance to the effects of these anabolic peptides [162]. The only available study in children with autoimmune hepatitis (AIH) showed no significant changes in ghrelin levels between the group with AIH and controls. This was probably due to the fact that patients with AIH had no clinical symptoms and predominantly low Pediatric End-Stage Liver Disease (PELD) or Model for End-Stage Liver Disease (MELD) scores [163].

Data on ghrelin concentration in CHC and chronic hepatitis B (CHB) are limited [164,165,166], including those for patients on antiviral treatment [167,168]. Plasma AG concentrations were significantly lower in relation to the severity of liver disease in both the HBV- and HCV-infected patients. In addition, liver cirrhosis and HCV infection were identified as independent factors associated with reduced AG levels [164]. Pavlidis et al. have observed that patients with genotype-1 HCV who achieved sustained virological response (SVR) had higher ghrelin concentrations at the baseline than non-responders. In patients with genotype-3 HCV, ghrelin may even be considered an independent factor, as responders with moderate to severe steatosis had high ghrelin levels at the baseline, and these levels decreased significantly after treatment. Studies suggest that ghrelin may prevent or reduce steatosis by negatively regulating leptin, thereby increasing the probability of achieving SVR [168].

At the tissue level, there was a non-significant trend toward higher hepatic expression of mRNA ghrelin in patients with NASH compared to those with steatosis and normal livers [169]. Another study showed overexpression of ghrelin transcripts in livers with NAFLD compared to the other groups (alcoholic hepatitis, HCV-infected livers, and controls). In addition, the whole CLD group showed a correlation with the expression of genes involved in fibrogenesis. *GHRL* transcripts were present in both hepatocytes and activated, freshly isolated from the liver, hepatic stellate cells [42].

Regarding the role of obestatin in NAFLD/NASH, there was a negative correlation between serum concentration of ghrelin and obestatin and overweight status, obesity, and MetS in NAFLD patients. Compared with the controls, patients with NAFLD had lower serum ghrelin and obestatin levels. Ghrelin and obestatin would be protective against hepatic steatosis and were correlated with a low risk of developing NAFLD. In contrast, the ghrelin/obestatin ratio was not correlated with NAFLD [170]. In another study, obestatin levels in NASH patients increased with the fibrosis stage [155]. There are also results of studies showing no differences in serum obestatin levels in patients with NAFLD and controls [171].

A summary of the serum levels/tissue expression of ghrelin and obestatin in the most common human liver diseases (including hepatitis) is presented in Table 3.

### 5.2. Genetic Study

Attempts have been made to study genetic variants in *GHRL* in various CLDs in humans in the context of their prognostic significance [42,165,166,175]. Moreno et al., studying European patients with CHC, observed that those with the -994T and -604A haplotype are more prone to severe liver fibrosis [42]. Of the three single nucleotide polymorphisms (SNPs) of *GHRL* in other study of CHC, only one SNP (Arg51Gln) showed significantly higher GA, AA genotypes, and A allele frequencies in patients who developed HCC compared to patients without HCC development and controls. These results suggest that the A allele at position 346 of *GHRL* is associated with susceptibility to HCC in CHC Egyptian patients [175]. In contrast, Zhang et al. showed that the *GHRL* rs26311 polymorphism may be a risk factor for HBV-related liver cirrhosis in a Chinese population, and mainly in men. These authors also observed an inverse correlation between serum ghrelin levels and liver cirrhosis [165]. Other authors confirmed significantly lower serum ghrelin levels in patients with CHC compared to controls. However, there was no significant association of the tested *GHRL* rs26312 and rs27647 polymorphisms with ghrelin levels in CHC patients. Neither of these SNPs of *GHRL* affect the response to combination treatment with sofosbuvir and simeprevir in patients with CHC [166].

### 5.3. Animal Models of Liver Inflammation

Numerous animal models were used to explain the effects of the ghrelin system on various forms of liver damage, including its role in inflammatory processes associated with NAFLD/NASH. Thus, chronologically, in rats with acetaminophen-induced liver injury, ghrelin has been shown to reduce the activities of hepatic enzymes aspartate aminotransferase (AST) and alanine aminotransferase (ATL), as well as reduce TNF-α levels [176]. In a rat model, recombinant ghrelin (eG) was also shown to reduce parameters of acute liver injury induced by carbon tetrachloride (CCl_4_) [42,177]. It was shown to reduce necroinflammatory score and AST serum levels, which was correlated with a decrease in inflammatory infiltration (CD43-positive cells) and the number of apoptotic cells in liver sections. The mechanism of the hepatoprotective effect of ghrelin was attenuation of NF-κB activation (p65 nuclear translocation), the effect of CCl_4_ on serine/threonine-protein kinase (Akt), and extracellular signal-regulated kinase (ERK) phosphorylation [42]. An identical research model showed decreasing histological alterations by ghrelin, reducing plasma and liver MDA content and plasma nitric oxide (NO) levels, as well as increasing superoxide dismutase (SOD), CAT, and GPx activities in erythrocytes and hepatic tissues compared to the CCl_4_ treated group. In addition, both ghrelin alone and ghrelin+CCl_4_ raised serum glucose levels [177].

Similarly, ghrelin’s downregulation of serum liver enzymes and TNF-α was confirmed in a rat model of chronic liver injury with thioacetamide treatment. Ghrelin decreased the expression of collagen, MDA, and Bcl-2-associated X protein (Bax) genes in liver tissue, and increased the expression of B-cell lymphoma 2 (Bcl-2) and endothelial nitric oxide synthase (eNOS) genes. Thus, according to the authors, the hepatoprotective effect of ghrelin is, at least in part, mediated by NO release [178]. Similar exponents of reduced liver damage were obtained by Mao et al. in concanavalin A-induced acute immune hepatitis in mice. These authors conclude that ghrelin impairs acute immune hepatitis by activating the phosphoinositide 3-kinase (PI3K)/Akt pathway and inhibiting the process of autophagy [179]. Ghrelin also causes an anti-inflammatory action through modulation of n-6 polyunsaturated fatty acid (PUFA) inflammatory pathways in liver tissue. In sodium metabisulfite-treated rats, ghrelin treatment reduced n-6 PUFA, COX, and PGE2 levels in the liver [180].

Studies on the effects of ghrelin in a rat model of NAFLD have shown that this peptide might attenuate NAFLD-induced liver injury, reduce inflammation and oxidative stress, and has an anti-apoptotic effect [34,41,84,181,182]. The main mechanisms are the serine-threonine liver kinase B1/AMP-activated protein kinase (LKB1/AMPK) and PI3K/Akt pathways [34]. Administration of AG reduced TG accumulation, and normalized tissue redox state and inflammation markers in diet-induced obese rats [41]. The protective effect of ghrelin was also demonstrated by Nagoya et al. in another animal model of NAFLD. They observed that the fatty changes in the liver stimulate autonomic neural signaling circuits, suppressing disease progression by activating gastric ghrelin expression and hepatic IGF-1 release [183].

In the context of NAFLD development, there has also been interest in the involvement of ghrelin in autophagy, the process with an intrinsic role in hepatic lipid metabolism [184]. In an HFD mouse model, Mao et al. described that ghrelin reduces TG content. In addition, TNF-α and IL-6 levels were significantly lower in ghrelin-treated mice compared to controls. In ghrelin-treated mice, the reduction in intracellular lipids was accompanied by induction of autophagy via AMPK/mTOR restoration. In addition, ghrelin inhibited NF-κB translocation into the cell nucleus [181].

Very interesting work by Ande et al. on a new model of obese transgenic mice (Mito-Ob) indicates that obesity alone is not sufficient for the development of NASH and HCC, but rather requires the additional influence of AT inflammation, hyperinsulinemia and chronic low-grade hepatitis. The authors showed sexual dimorphism in ghrelin levels in female mice (significantly elevated) vs. male mice (reduced) compared to control mice, while Mito-Ob males had significantly reduced ghrelin levels compared to WT mice. Livers with tumors from male Mito-Ob mice showed significantly reduced ghrelin levels compared to livers from mice without tumors and to WT mice. Thus, factors such as inflammation of AT, reversal of serum insulin and ghrelin levels, increase in hepatic lipid accumulation, infiltration of macrophages and lymphocytes, and reduction in hepatic mitochondrial content and function, with a concomitant increase in hepatic DNA damage, cell death, and compensatory proliferation, are important in the progression of HCC [185].

It has also been proven in animal models that bariatric surgery (sleeve gastrectomy in obese rats) improves NAFLD. The gastrectomy procedure induced a significant decrease in UnAG concentrations, but increased the AG/UnAG ratio. In addition, gastrectomy decreased hepatic TG content and the monoacylglycerol O-acyltransferase-1 and diacylglycerol O-acyltransferase-1 (Mogat2 and Dgat1) (lipogenic enzymes), increased mitochondrial DNA, and induced AMPK-activated mitochondrial β-oxidation of free fatty acid (FFA) and autophagy to a greater extent than caloric restriction. These studies suggest that a decrease in UnAG after gastrectomy reduces lipogenesis, while the increased relative AG levels activate factors involved in mitochondrial FFA β-oxidation and autophagy in obese rats, i.e., improve NAFLD [84]. The same team of researchers confirmed that the increase in relative levels of AG after bariatric surgery in rats may help to alleviate obesity-related hepatitis, mitochondrial dysfunction, and endoplasmic reticulum (ER) stress [87]. Another rat model of NAFLD (HF-high-cholesterol diet) showed decreased values of plasma total ghrelin, UnAG, and the UnAG/AG ratio, with increased expression (protein, mRNA) of hypothalamic AG and GHS-R1a. The authors suggest that an imbalance of circulating UnAG/AG, and not the values of AG or UnAG alone, might be involved in insulin resistance (IR) and lipid accumulation in NAFLD. Moreover, the data indicate that UnAG appears to lower TGs as suggested by a negative correlation between these variables. AG via a central mechanism in the hypothalamus is supposed to be responsible for inducing IR and promoting lipid accumulation [182]. Recently, in HFD-fed rats, it was shown that lowering AG levels, as well as reducing AG/UnAG ratio by exogenous administration of UnAG, alleviates hepatic steatosis (and thereby NAFLD) by inhibiting lipogenesis, stimulating FFA oxidation, and preventing oxidative stress, inflammation, ER stress, and apoptosis [186]. Finally, a mouse model of hepatic ischemia-reperfusion injury (HIRI) causing acute-on-chronic liver failure was established to examine the effects of liver injuries transformed to liver fibrosis. The authors confirmed the protective function of ghrelin on the liver (e.g., improving histopathological changes, lowering plasma ALT, reducing MPO expression, and exerting an anti-apoptotic and antioxidant effects) [37].

Considering that ghrelin enhances obesity and hepatic steatosis in rodents and humans with age [28], the mechanisms of this process have also been studied in a ghrelin knockout (KO) mouse model. It has been shown that ghrelin KO mice lack the increase in expression of Dgat1 (observed with age in WT mice), one of the key enzymes of TG synthesis. This is due to the lack of activation of CCAAT/enhancer binding protein-alpha (C/EBPα) and the subsequent reduction in C/EBPα-p300 complexes, as well as the lack of activation of the *DGAT1* promoter in ghrelin KO mice. Interestingly, the mechanism by which ghrelin deletion prevents age-related hepatic steatosis suggests that targeting this pathway may have therapeutic benefits in NAFLD [187].

In contrast with the protective effect of ghrelin on the liver with inducible NAFLD, studies in non-obese animals (lean rats fed on standard diet) have shown that administration of exogenous AG carries a high risk of developing steatohepatitis and hepatic IR, while the addition of a balanced dose of UnAG reduces this risk, inhibits hepatic lipid accumulation, and enhances hepatic insulin signaling. These actions occurred involving AMPK/PPAR-α/carnitine palmitoyltransferase-1 (CPT-1) signaling inhibition. Consequently, AG induced membrane translocation of PKCδ and PKCε leading to JNK activation and significant inhibition of insulin signaling under basal conditions and upon insulin stimulation [188].

As for the role of obestatin, in an animal model (rats) with HIRI model, it was observed that obestatin counteracted hepatic injury mainly by reducing oxidative stress, inhibiting the proinflammatory cytokines (TNF-α, and IL-6), and modulating NO levels [189]. It has been suggested that obestatin also reverses and protects against the development or progression of NAFLD directly, by modulating ghrelin and adiponectin signaling, or indirectly, by reducing food intake. Obestatin treatment resulted in decreased hepatomegaly, reduced hyperlipidemia, hepatic lipid accumulation, and IR. Obestatin increased circulating adiponectin levels and hepatic signaling (increased levels of hepatic adiponectin receptors (adipoRII), CPT-1, PPAR-α, and p-AMPK). In addition, obestatin increased total circulating levels of ghrelin and significantly increased the UnAG/AG ratio [190].

The anti-fibrotic effects of ghrelin have also been demonstrated, which may have clinical implications [42,178,191]. The role of ghrelin in inhibiting liver fibrosis occurs mainly through reducing collagen deposition [42,178], α-smooth muscle actin (α-SMA) expression, and lowering the accumulation of myofibroblastic fibrogenic cells. Ghrelin treatment also attenuated changes in the expression of 231 genes, including collagen-α1 (II). In mice *ghrl^(−/−)^*-induced to liver fibrosis, overexpression of procollagen-α2 (I) and tissue inhibitor of metalloproteases (TIMP-1), as compared with *ghrl^(+/+)^* mice, was observed. In this regard, inhibition of fibrosis in the livers of rats was observed by using the agonist GHS-R, as indicated by decreased collagen deposition [42]. The study by Mao et al. confirmed a reduction in pathological lesion extent, collagen-I, and α-SMA expression after ghrelin administration in two models of liver fibrosis in C57BL/6 mice. These studies suggest that ghrelin attenuates liver fibrosis by inhibiting TGF-β1/Smad3 and NF-κB signaling pathways, as well as inhibiting autophagy [191]. These data were also confirmed by a recent study by Yang et al., who demonstrated inhibition of HSCs activation and blocking of classic fibrotic Smad and ERK signaling pathways by ghrelin. Reduction in liver fibrosis was achieved by stimulating the degradation of extracellular matrix (ECM) components such as collagen-I, collagen-III, hyaluronic acid, and laminin [37].

### 5.4. In vitro Models of Liver Inflammation

Although they are very few, there are also papers on in vitro models that point to a role for ghrelin in hepatic lipid metabolism [36,40,84,181], inflammatory injury, and fibrosis [36,42,86].

In the aforementioned work on a model of HFD-induced obese mice, ghrelin via GHS-R1a was shown to block the progression of LPS-induced NASH by attenuating M1 polarization of Kupffer cells. In addition, ghrelin decreased TNF-α and inducible NOS (iNOS) (M1 marker) levels while increasing Arg-1 levels (M2 marker) in LPS-treated Kupffer cells. The peptide attenuated lipid accumulation induced by Kupffer cell supernatants both under basal conditions and under LPS treatment [36]. Interesting in vitro studies also followed the animal model experiments performed by Ezquerro et al. [84]. Studies on primary rat hepatocytes showed that both AG and UnAG increased mRNA expression of lipogenic genes: *MOGAT2*, *DGAT1* and intracellular TG content. Both isoforms of ghrelin were shown to enhance AMPK and acetyl-CoA carboxylase (ACC) phosphorylation rates, modulating mitochondrial FFA β-oxidation. Moreover, AG increased the expression of autophagy-related markers, as ATGs and the microtubule-associated protein light chain 3 II/I (LC3B-II/I) ratio, while p62 (a classical receptor of autophagy) levels were markedly reduced. In contrast, UnAG only modified the expression of p62. The study suggests that AG and, to a lesser extent, UnAG, stimulate hepatic autophagy [84], whereas ghrelin treatment of normal human hepatocytes (LO2 cells) exposed to FFAs showed a reduction in TG content in these cells, and was associated with an increase in autophagy. Ghrelin promoted autophagy partly via restoration of the AMPK/mTOR signaling pathway. In addition, the FFA+ghrelin group had higher levels of NF-κB in the cytoplasm and a lower level in the cell nucleus. These studies provide evidence that ghrelin attenuated inflammatory liver injury by NF-κB inhibition (translocation to the cytoplasm) and autophagy enhancement [181]. Continuing their research in an in vitro model as well, Moreno et al., using both unstimulated and angiotensin II-stimulated HSCs, showed an inhibitory effect of ghrelin on liver fibrogenesis. A reduction in collagen-α1 (I) and TGF-β synthesis was observed in HSCs exposed to ghrelin. In contrast, no inhibition of proinflammatory actions of HSCs was observed [42].

There are also few results of studies on the effects of ghrelin on hepatic lipid metabolism, conducted on human HCC cells. Thus, using HepG2 cells, administration of AG and UnAG reduced TNF-α-induced cell apoptosis, as well as pyroptosis. In addition, AG suppressed TNF-α-activated hepatocyte autophagy, as evidenced by a reduced LC3B-II/I ratio and increased AMPK/mTOR-mediated p62 accumulation [86].

There are new cell culture models for viral hepatitis which are primarily for the analysis of the molecular mechanisms of HBV/HCV infections, the discovery of antiviral drugs to ultimately eradicate chronic infections, and/or the development of effective vaccines [192,193,194]. So far, the effects of ghrelin have not been studied in these models of hepatitis.

In conclusion, in vitro studies have clarified the mechanisms of beneficial effects of both ghrelin isoforms in various models of liver inflammation and fibrosis. Disturbances in the regulation of these processes may play a role in the progression of liver injury to HCC. The peripheral effect of ghrelin on hepatic lipid metabolism has been controversial. In vitro studies in rats and mice have shown that ghrelin enhances lipogenesis by increasing TG content and lipogenesis genes in cells, via the mammalian target of rapamycin/peroxisome proliferator-activated receptor γ (mTOR)/PPARγ) signaling pathway [40,84], while normal human hepatocytes show a decrease in lipid accumulation after ghrelin treatment, which appears to be related to an increase in autophagy and a decrease in mTOR phosphorylation [181].

The main findings on ghrelin effects in animal models and in vitro studies of different types of liver injury (including NAFLD) are presented in Table 4.

There is a lack of in vitro studies on the effect of obestatin in inflammation of the liver promoting and/or inhibiting the development of HCC.

## 6. Ghrelin System in the Treatment of Inflammatory Bowel and Liver Diseases

So far, experimental work on the effects of treatment with exogenous ghrelin and obestatin in IBDs has been performed on animal models of colitis, showing mainly anti-inflammatory and cytoprotective systemic/local effects of both peptides (Table 1). However, none of the components of the ghrelin system are currently used in the treatment of IBDs in humans. One of the reasons is the occurrence of increasingly innovative forms of anti-inflammatory therapy and more effective methods of combating inflammation in the intestine [195,196]. However, there are interesting observations on the protective effect of flavonoids on IBD regulating the activity of enterohormones, including the ghrelin pathway [197].

Most studies on various animal models of hepatitis also point to a hepatoprotective role of exogenous ghrelin through a reduction in histopathological changes and inhibition of the inflammatory process, as well as antioxidant, anti-apoptotic, and anti-fibrotic effects. Conflicting data concern the effects of ghrelin on hepatic lipolysis (enhancing, inhibiting), including the possibility of exacerbating NAFLD (Table 4). These observations make it difficult to promote ghrelin alone for the treatment of NASH in humans, pointing to other therapeutic options [10]. To date, there is no recommendation for treatment with any component of the ghrelin system in liver disease. Well known applications for ghrelin and its derivatives (ghrelin analogs, and GHS-1Ra agonists) at present, are primarily cancer cachexia and sarcopenia [198,199]. Clinical studies have also been undertaken on ghrelin treatment in cachexia in chronic heart failure, chronic obstructive pulmonary disease, and end-stage renal disease or cystic fibrosis, but also in GI motility disorders, after curative gastrectomy, anorexia nervosa, in patients with GH deficiency, on alcohol starvation, in the sleep–wake regulation (such as in major depression), or on sympathetic nervous activity in obesity [200]. The ghrelin agonists (renamorelin) have recently been mentioned among prokinetics for use in patients with chronic constipation [201].

## 7. Final Remarks and Future Perspectives

Understanding the role of hormonal and metabolic factors in cancer-related inflammation may help to provide insight into the still poorly understood mechanisms of the transition from chronic inflammation to cancer. The results of clinical studies on the role of the ghrelin system in inflammatory GI tract diseases (e.g., IBDs, non-alcoholic steatohepatitis, viral and autoimmune hepatitis, and liver cirrhosis), as well as their progression to CRC or HCC, are difficult to interpret. In the case of IBD, there is generally an increase in ghrelin serum levels/tissue expression, especially in active forms of UC and CD, compared to patients in remission (Table 1).

In liver disease, depending on the etiology, changes in serum ghrelin levels are even more variable compared to IBD. In NAFLD and liver cirrhosis, both decreases and increases in ghrelin (AG and UnAG) were observed. In HBV/HCV-associated hepatitis, it is mainly a decrease in ghrelin (including AG) levels that is observed. Changes in ghrelin concentrations vary depending on the severity of organ fibrosis (increase, decrease). In HCC of different etiologies, an increase in ghrelin levels was observed compared to the controls (Table 3). It is difficult to determine the primary cause of changes in serum ghrelin/obestatin concentrations in clinical studies. It cannot be ruled out that these fluctuations are a feedback response to changes in nutrient absorption, gastrointestinal function, caloric intake, or other unknown factors (e.g., bacterial flora). It is also a possible scenario that the increase in endogenous ghrelin in inflammatory gastrointestinal diseases plays a cytoprotective role in colitis and hepatitis, compensating for the effects caused by local/systemic proinflammatory factors. On the other hand, serum ghrelin levels in IBD and liver disease, correlated with other inflammatory markers in patients, could result in worsening of the local inflammatory process.

**Figure 2:**
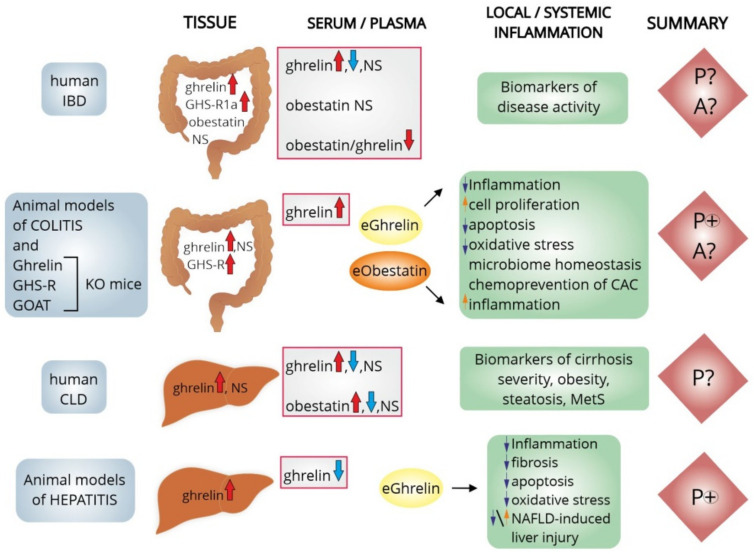
Potential role of the ghrelin system in the local and systemic inflammatory process of the colon and liver in humans and in various animal models of colitis and hepatitis. Protective, anti-inflammatory, and healing effects are the main effects of exogenous ghrelin and obestatin in animal models of colitis, however, there are some experimental data showing the proinflammatory effects of ghrelin in mice models of colitis. In chronic inflammatory liver diseases of various etiologies, ghrelin also exhibits hepatoprotective (anti-inflammatory, antioxidant and anti-fibrotic) effects. The protective effects of ghrelin have also been shown in various animal models of NAFLD, although enhancement of lipogenesis and lipid accumulation in the liver of mice after administration of exogenous ghrelin has also been described. The protective role of the ghrelin system in colitis/hepatitis-associated carcinogenesis is still unclear and requires continued research. [↑/↓—increase/decrease; A?—possible aggravating effect on inflammation; CAC—colitis-associated cancer; CLD—chronic liver disease; eGhrelin—exogenous ghrelin; eObestatin—exogenous obestatin; GHS-R(1a)—ghrelin receptor (1a); GOAT—ghrelin-O-acyltransferase; IBD—inflammatory bowel disease; KO—knockout; MetS—metabolic syndrome; NAFLD—nonalcoholic fatty liver diseases; NS—non-significant expression/level; P(+)—definite protective role; P?—most likely protective effect].

Studies in animal models and in cultured cells primarily confirm the protective role of the ghrelin system in the colon and liver. In both of these organs, administration of exogenous ghrelin/obestatin reduced inflammation-induced organ damage. Thus, it had a hepatoprotective role in the chronically injured liver (Figure 2). However, the exact role of ghrelin in the progression of chronic inflammatory lesions into cancer is still unknown. Further research is required to assess the direct mechanisms of action of ghrelin/obestatin in inflammatory-related premalignant lesions of the colon and liver. The introduction of drugs based on the action of the ghrelin system, or the modification of its beneficial effects on IBDs and inflammatory liver diseases, requires continued research.

## 8. Conclusions

Table 5 summarizes the main anti-inflammatory effects and the immunomodulatory potency of the ghrelin system in various models of colon and liver inflammatory diseases.

## Figures and Tables

**Table 1:** The Ghrelin System Components in the Colon Inflammation—Animal and in vitro Studies.

Model of the Study	Material and Methods	Tissue Expression		The Main Effects of Ghrelin/Obestatin	Mechanisms of Action/Pathways; Role in CRC Development	Ref.
		Ghrelin/Obestatin	GHS-R			
Animal	mice; TNBS/DSS colitis; eG 1 nmol/mouse/d	nt	nt	(i) ↓ acute and chronic inflammatory response; (ii) ↓ of both inflammatory and Th1-driven autoimmune response; (iii) prevents recurrence of the disease; (iv) ↓ NF-κB	Anti-inflammatory; NF-κB; role in cancer—nd	[38]
	rats; TNBS colitis and C; eG 20 µg/kg	↑ mRNA (max at day 7th)	nt	(i) ↑ healing of colonic lesions with ↑ mRNA (iNOS, PGE2) and protein (COX-2); (ii) ↑ of neuropeptides (e.g., CGRP) from sensory nerves	Anti-inflammatory; role in cancer—nd	[39]
	mice; *C. rodentium*-colitis; 7-plex base kit; RT-PCR; no eG treatment	↑ mRNA at the peak and late stage	nt	(i) in the distal colon: ↑ TNF-α and FoxP3 throughout the study; (ii) ↑ IL-6 and IL-17 during the peak and late stages of infection	Anti-inflammatory—probable; Th cell pathways; G can be involved with clearance of infection; role in cancer—nd	[138]
	rats; DSS colitis; eG 20 µg/kg	nt	nt	(i) ↓ severity of chronic colitis, less effective in the acute form; (ii) ↓ lipid peroxidation and Th1 response; (iii) in chronic colitis: ↓ of pro- and ↑ of anti-inflammatory cytokines (TGF-β)	Anti-inflammatory; role in cancer—nd	[115]
	rats; DSS colitis; eO 50 µg/kg	nt	nt	(i) ↓ disease activity in acute and in chronic colitis; (ii) ↓ MDA; (iii) ↑ GSH; (iv) in acute colitis: ↓ IL-1β and TNF-α in colon; (v) in chronic colitis: ↓ IL-1β, IFN-γ, TNF-α with ↑ IL-10 and TGF-β in colon	Anti-inflammatory; role in cancer—nd	
	rats; AA colitis and C; eG 4–16 nmol/kg/dose	nt	nt	(i) ↓ the area and grade of mucosal damage; (ii) ↓ IL-1β and MDA in mucosa; (iii) ↓ MPO activity	Anti-inflammatory; role in cancer—nd	[142]
	rats; DSS colitis and C; eG 8 nmol/kg/dose	nt	nt	(i) ↓ of mucosal damage; (ii) ↓ IL-1β and MDA in mucosa; (iii) reversed ↓ in BW gain	Anti-inflammatory; role in cancer—nd	[144]
	mice; AOM/DSS colitis; (*Apc^(Min/+)^* model; eG; RT-PCR, IHC	nt	nt	(i) ↓ in tumor incidence in AOM/DSS colitis; (ii) no tumor-promoting effect in either model; (iii) loss of G did not affect the incidence of intestinal tumor formation in either model	The chemopreventive effect of inflammation-associated colorectal carcinogenesis	[148]
	mice; DSS colitis; eG 125 or 250 μg/kg	nt	nt	(i) ↓ the disease activity index, histological score, and MPO activities; (ii) ↑ in TJ structural integrity and cytokine secretion; (iii) ↓ NF-κB, inhibitory κB-α, MLCK, and pMLC2 activation	Anti-inflammatory; GHS-R1a; NF-κB; role in cancer—nd	[143]
	mice; T cell transfer model of chronic colitis; *Rag^(−/−)^* mice; eG 0–100 ng/mL; FC	nt	nt	(i) the lack of G signaling in Th cells resulted in a ↑ severity of colitis with ↑ colonic inflammation dependent on a pathological ↑ of CD4 T cells in lamina propria; (ii) ↓ proliferation and ↑ apoptosis of Th cells; (iii) specific effect on Th cells	Anti-inflammatory; role in cancer—nd	[149]
	rats; AA-induced colitis and C; eAG 8 nmol/kg/dose	nt	nt	(i) ↓ histological colonic damage and ↑ spontaneous colonic regeneration; (ii) ↑ DNA synthesis in mucosa; (iii) ↑ of blood flow in mucosa; (iv) ↓ IL-1β, TNF-α, and MPO activity in mucosa	Anti-inflammatory; role in cancer—nd	[145]
	rats; AA colitis and C; rat O 8 nmol/kg; 2×/day; 7 and 14 days	nt	nt	(1) ↑ healing of colonic lesions; (ii) ↓ MPO and IL-1β in mucosa; (iii) reversed the colitis-evoked decrease in blood flow and DNA synthesis (↑ cell proliferation in mucosa)	Anti-inflammatory; role in cancer—nd	[116]
	rats; AA colitis and C; eAG 8 nmol/kg/dose	nt	nt	(i) ↓ damage of mucosa only in pituitary-intact rat, correlated with ↑ serum levels of GH and IGF-1; (ii) ↑ blood flow and ↑ cell proliferation; (iii) ↓ IL-1β, MDA and MPO activity in mucosa	Anti-inflammatory; GH and IGF-1; role in cancer—nd	[107]
	rats; AA colitis; eO 4, 8 or 16 nmol/kg/dose; 2×/day	nt	nt	(1) ↓ the area of colonic damage; (ii) ↑ blood flow and DNA synthesis in mucosa; (iii) dose-dependent ↓ IL-1β and MPO in mucosa	Anti-inflammatory; role in cancer—nd	[118]
	rats; TNBS colitis and C; eO 4, 8, or 16 nmol/kg, 2×/day, 4 days	nt	nt	(1) dose-dependent ↓ the area of colonic damage; (ii) ↑ blood flow in the colon; (iii) ↓ MPO activity and IL-1β in mucosa; (iv) ↓ blood leukocytes	Anti-inflammatory; role in cancer—nd	[117]
	(*Ghsr*^−/−^) mice; DSS colitis; no eG treatment	nt	nt	(i) in GHS-R KO mice: ↑ disease activity scores, ↑ expression of TNF-α, and IL-1β, and ↓ expression of TJ markers (occludin, claudin 2); (ii) ↑ gut permeability and exacerbated colitis	Anti-inflammatory; role in microbiome homeostasis and gut inflammation during aging; role in cancer—nd	[141]
	mice; DSS colitis; *GOAT^(−/−)^* mice; no eG treatment	nt	nt	KD of GOAT: ↓ colitis-induced inflammation and ↓ apoptosis by ↓ the intestinal permeability; (ii) GOAT overexpression: ↑ colitis	Proinflammatory role of GOAT; role in cancer—nd	[72]
	mice; DSS and TNBS colitis; eG 25–250 µg/kg	nt	nt	(i) protection from apoptosis; (ii) ↓ apoptosis in a dose-dependent manner, reversed by D-Lys3-GHRP-6	Anti-apoptotic; GHS-R1a/GHS-R1b; UPR; role in CRC—nd	[146]
	mice; TNBS colitis; no eG treatment; RT-PCR	↑ mRNA	↑ mRNA	↑ G and GHS-R during acute experimental colitis	Proinflammatory probable; role in cancer—nd	[139]
	G^(+/+)^ and G^(−/−)^ mice; DSS colitis; eG 100 nmol kg^−1^	nt	nt	(i) ↑ in G^(+/+)^ plasma levels; (ii) ↑ clinical disease activity, ↑ infiltration of neutrophils, and ↑ colonic IL-1β levels; (iii) absence of G did not affect colonic contractility; (iv) in G^(−/−)^ mice: ↓ BW loss, ↓ histological damage, ↓ MPO, IL-1β levels	Proinflammatory; role in cancer—nd	[147]
	WT-mice and (*Ghsr*^−/−^) mice; DSS colitis; no eG treatment	no changed at day 7th	↑ mRNA at day 7th	(i) ↓ colonic macrophage infiltration and TLRs expression from DSS-treated (*Ghsr*^−/−^) mice vs. WT-mice	Proinflammatory; role in pathogenesis of IBD—probable, role in cancer—nd	[140]
In vitro	normal human colon NCM460 cells transfected with a functional GHS-R; eG 10^−9^–10^−7^ M	nt	(+) mRNA	(i) ↑ IκBα phosphorylation and degradation; (ii) stimulation of NF-κB-binding activity and NF-κB p65 subunit phosphorylation; (iii) ↑ TNF-α-induced IL-8 promoter activity and IL-8 protein secretion	Proinflammatory; PKC-dependent NF-κB; role in cancer—nd	[139]
	NCM460 cells transfected with GHS-R; eG 10^−8^ M	nt	nt	(i) ↑ COX-2 protein/promoter activity; (ii) ↑ PGE2 secretion; (iii) ↑ phosphorylation of CREB via PKCδ activation; (iv) ↑ phosphorylation of PKCδ	Proinflammatory; PKC; role in cancer—nd	[151]
	T cell transfer model of chronic colitis; eG 0–100 ng/mL	nt	nt	(i) directly affected Th cells: ↓ proliferation and ↑ apoptosis; (ii) did not influence Th cell polarization	Regulation of Th cells in gut, anti-proliferative and anti-apoptotic role in IBD—probable; role in cancer—nd	[149]
	MA from (*Ghsr*^−/−^) LPS-stimulated mice; MA from WT mice; DSS colitis; IHC; ELISA	nt	nt	(i) ↓ IL-6, TNF-α, IL-1β, TLR-2, TLR-4 levels in MA from (*Ghsr*^−/−^) mice vs. WT-mice; (ii) D-lys(3) -GHRP6: ↓ LPS-induced MA proinflammatory cytokines from WT mice	GHS-Rs: role in acute colitis and MA activation in vitro; role in pathogenesis of IBD—probable, role in cancer—nd	[140]
	human colon HCT116 cells; eG 10^−8^ M	nt	nt	(i) ↓ ROS via ↑ activity of CAT and MnSOD vs. untreated cells; (ii) ↓ MDA (G + leptin)	Anti-inflammatory; role in pathogenesis of IBD—probable, role in cancer—nd	[150]
	TNF-α-induced Caco-2 cells; eG 0.01–10 µmol/L; RT-PCR, WB	nt	nt	(i) ↓ apoptosis	Anti-apoptotic; GHS-R1a; UPR; role in cancer—nd	[146]

[↑/↓—increase/(stimulation)/decrease (inhibition); (+)—positive expression; AA—acetic acid; AG—acylated ghrelin; AOM—azoxymethane; Apc*^(Min/+)^*—adenomatous polyposis coli/multiple intestinal neoplasia^+^; BW—body weight; C—control; CAT—catalase; COX-2—cyclooxygenase-2; CREB—cAMP responsive element-binding protein; d—day; DSS—dextran sodium sulphate; (e)G—(exogenous/recombinant) ghrelin; (e)O—(exogenous) obestatin; FC—flow cytometry; FoxP3—forkhead box P3; GH—growth hormone; GHS-RA—ghrelin receptor type 1a antagonist (D-Lys3-GHRP-6); GHS-R1a/1b—GH secretagogues receptors 1a/1b; GOAT—ghrelin-O-acyltransferase; GSH—glutathion; IBD—inflammatory bowel diseases; IGF-1—insulin-like growth factor 1; IFN-γ—interferon gamma; IL-1β; -10—interleukin 1β, IL-10; iNOS—inducible nitric oxide synthase; IHC—immunocytochemistry; IκBα—inhibitory protein of IκB family; KD/KO—knockdown/knockout; LPS—lipopolysaccharide; MA—macrophages; MDA—malondialdehyde; MLCK—myosin light chain kinase; MnSOD—manganese superoxide dysmutase; MPO—myeloperoxidase; nd—not determined/non-available data; NF—κB-nuclear factor κB; nt—non-tested; PGE2—prostaglandin E2; PKCδ—protein kinase C delta; pMLC2—phosphorylated myosin light chain 2; ref.—number of reference; RT-PCR—real-time polymerase chain reaction; TGF-β—transforming growth factor beta; Th1—T helper cell; TJ—tight junction; TLR—toll-like receptors; TNBS—trinitrobenzene sulphonic acid; TNF-α—tumor necrosis factor-alpha; UPR—unfolded protein response; WB—Western blotting; WT—wild type].

**Table 2:** Summary of the Role of Ghrelin and Obestatin in Different Models of Inflammatory Bowel Disease.

IBD Colitis	Ghrelin	Refs.	Obestatin	Refs.
Human IBD	A	[122,123,124,125,126,127,128]	A	[125]
	P	[129]	P	[128]
	NS	[130]	nd	
DSS	P	[38,115,141,143,146]	P	[115]
	A	[140,147]	nd	
TNBS	P	[38,39]	P	[117]
AA	P	[107,142,145]	P	[116,118]
*C. rodentium*	P	[138]	nd	
AOM/DSS	P	[148]	nd	

[A—aggravating role; AA—acetic acid; AOM—azoxymethane; C—*Citrobacter*; DSS—dextran sodium sulphate; IBD—inflammatory bowel disease; nd—non-available data; NS—non-significant impact; P—protective role; ref.—number of reference; TNBS—trinitrobenzene sulphonic acid].

**Table 3:** Serum Levels/Tissue Expression of Ghrelin and Obestatin in the Most Common Human Liver Diseases.

Liver Disease	Ghrelin	Refs.	Obestatin	Ref.
NAFLD/NASH	↓ AG vs. C	[153,154,170]	↓ O in NAFLD vs. C	[170]
	↑ UnAG vs. non-NASH and with more advanced fibrosis	[155]	↑ O with fibrosis stage	[155]
	NS trend to ↑ G tissue expression vs. nonalcoholic steatosis and C	[169]	NS O vs. C	[171]
	↑ G mRNA vs. alcoholic hepatitis, HCV-infected livers, and C	[42]		
Chronic hepatitis B	↓ AG vs. C	[164]	nd	
Chronic hepatitis C	↓ AG vs. C	[164]	nd	
	↓ G vs. C	[42]		
Alcoholic hepatitis	↓ G vs. C	[42]	nd	
Cirrhosis (different etiologies)	↑ G in Child C cirrhosis vs. CLD with no cirrhosis	[158]	nd	
	↑ G vs. C	[172]		
	↓ G in advanced vs. mild fibrosis	[42]		
	↑ AG vs. C	[162]		
	↓ AG and ↑ UnAG associated with cirrhosis severity	[160]		
	↓ AG in viral-associated cirrhosis vs. C	[164]		
	↓ G vs. C	[161]		
	↑ G in PBC vs. C	[173]		
HCC (different etiologies)	↑ G vs. C	[172]	nd	
Autoimmune hepatitis	NS G vs. C	[163]	nd	
Acute hepatitis	↑ G vs. liver after recovery	[174]	nd	

[↑/↓—increase/decrease level, expression; AG—acylated (active) ghrelin; C—control; normal liver; CLD—chronic liver diseases; G—ghrelin (type—nd); HCC—hepatocellular carcinoma; HCV—hepatitis C virus; NAFLD—non-alcoholic fatty liver disease; NASH—non-alcoholic steatohepatitis; nd—non-determined/non-available data; NS—non-significant; O—obestatin; PBC—primary biliary cirrhosis; ref.—number of reference; UnAG—unacylated (non-active) ghrelin].

**Table 4:** Role of Ghrelin in Various Types of Liver Injury—the Animal and in vitro Study Models.

Model of the Study	Material and Methods	The Main Effects of Ghrelin	Role in Inflammation/ Signaling Pathway	Ref.
Animal	rats; Acetaminophen-induced ALI; eG	(i) ↓ ALT and AST; (ii) ↓ TNF-α	P	[176]
	rats; CCl_4_-induced ALI and BDL-induced CLI; WT and G-deficient mice; eG	(i) ↓ necroinflammatory score and ↓ AST; (ii) ↓ inflammatory infiltration; (ii) ↓ apoptosis; (iii) ↓ fibrogenic response and ↓ fibrogenic properties of HSCs (iv) ↓ myofibroblasts accumulation; (v) ↓ extent of OS; (vi) altered gene expression profile in CLI; (vii) G-deficient mice: ↑ fibrosis and damage after CLI	P; Akt/ERK	[42]
	rats; CCl_4_-induced ALI; eG	(i) ↓ plasma/liver MDA, and NO level; (ii) ↑ erythrocyte/hepatic SOD, CAT and GPx; (iii) G alone and G+CCl_4_: ↑ glucose level; (iv) ↓ histopathological changes	P	[177]
	rats; TAA-induced CLI; eG	(i) ↓ ALT, AST and TNF-α levels; (ii) ↓ collagen in liver; (iii) ↓ MDA and Bax gene expression; (iv) ↑ Bcl-2 and eNOS gene expression	P; NO	[178]
	mice; concanavalin A-induced ALI; eG	(i) ↓ proinflammatory cytokines (IL-1β, IL-6, and TNF-α); (ii) ↑ Bcl-2, ↓ Bax, and ↓ caspases expression	P; PI3K/Akt/Bcl-2; autophagy	[179]
	mice; CCl_4_-and BDL-induced liver fibrosis; eG	(i) ↓ AST and ALT; (ii) ↓ histopathological changes in both models; (iii) ↓ collagen-I and α-SMA; (iv) ↓ protein expression of TGF-β and p-Smad3; (v) ↓ protein of NF-κB and LC3 in both models; (vi) ↓ ECM formation	P; TGF-β1/Smad3 and NF-κB; autophagy	[191]
	rats; sodium metabisulfite (Na_2_S_2_O_5_)-induced liver damage; rat G	↓ n-6 PUFA levels and ↓ COX and PGE2 levels in liver tissue	P; n-6 PUFA	[180]
	rats; HFD-induced NAFLD; eG	(i) ↓ ALT and AST and ↑ hepatic lipid metabolism; (ii) ↓ formation of OS; (iii) ↓ proinflammatory cytokines and apoptotic cells in the liver	P; LKB1/AMPK and PI3K/Akt	[34]
	rats; HFD-induced NAFLD; eG (AG)	(i) ↓ TG with concomitant ↑ GPx; (ii) normalized redox state and inflammatory markers (NF-κB and TNF-α)	P; NF- κB; Akt/AMPK	[41]
	mice; HFD-induced NAFLD; eG (AG)	(i) ↓ TG; (ii) ↓ TNF-α and IL-6	P; AMPK/mTOR; NF-κB; autophagy	[181]
	rats; HFD-induced NAFLD; sleeve gastrectomy; no eG treatment	After gastrectomy: (i) ↓ UnAG, ↑ AG/UnAG ratio; (ii) ↓ hepatic TGs and lipogenic enzymes Mogat2 and Dgat1; (iii) ↑ mDNA	P; gastrectomy: ↑ AMPK-activated mFFA β-oxidation and autophagy	[84]
	rats; HF-high-cholesterol diet-induced NAFLD; no eG treatment	(i) ↑ serum levels of TC, TGs, AST, ALT, hepatic TGs in NAFLD vs. C (ii) ↓ serum UnAG, total G, and the UnAG/AG ratio; (iii) ↑ hypothalamic AG and GHS-R1a	A; AG might induce IR and promote lipid accumulation via central mechanism	[182]
	mice; choline-deficient defined l-amino-acid diet-fed-induced NAFLD, melanocortin 4 receptor KO mice; partial hepatectomy mice with/without the blockades of autonomic nerves; no eG treatment	(i) ↑ gastric ghrelin expression through the autonomic pathways; (ii) ↑ GH in pituitary gland; (iii) ↑ hepatic IGF-1; (iv) high levels of ghrelin expression in the arcuate nucleus were correlated with NAFLD progression regardless of the circuits	P; GH/IGF-1	[183]
	rats; HFD-induced NAFLD; eG (UnAG)	(i) ↓ glucose level; (ii) ↓ serum/hepatic cholesterol, TGs, and FFA; (iii) ↓ levels of ROS, lipid peroxides; (iv) ↓ TNF-α and IL-6; (iv) ↓ Bax and caspase-3; (v) ↑ GSH, SOD, and Bcl-2	P	[186]
	mice; WT *G^(+/+)^;* G KO mice; no eG treatment	G^−/−^ mice: (i) lack of activation of C/EBPα resulted in ↓ of C/EBPα-p300 complexes and ↓ levels of DGAT1 and ↓ TGs	P; C/EBPα-/p300/DGAT1	[187]
	mice; HIRI-induced acute-on-chronic liver failure; eG	(i) ↓ histopathological changes; (ii) ↓ ALT, ↓ MPO expression; (iii) anti-apoptotic and antioxidant effects; (iv) ↑ ECM degradation	P; blocked fibrotic Smad and ERK	[37]
Animal/ In vitro	mice; HFD-induced obese mice, and db/db mice; GHS-R1a KO mice; hepatocytes from WT mice and HepG2 cells; eG	(i) GHS-R antagonism and KO of the gene: ↓ hepatic steatosis by ↓ de novo lipogenesis: (ii) eG: ↑ lipogenesis with ↑ TG in liver, ↑ hepatic lipid accumulation in mice; (iii) in vitro: ↑ lipogenesis via ↑ GHS-R1a activation with ↑ S6 protein	A; GHS-R1a mediated lipogenesis: mTOR/PPARγ	[40]
	rats; HFD-induced NAFLD; sleeve gastrectomy and RYGB; primary rat hepatocytes under palmitate-induced lipotoxic conditions; eG	After gastrectomy and RYGB: (i) ↓ UnAG, ↑ AG/UnAG ratio; (ii) both strategies: ↓ obesity-associated hepatic steatosis; (iii) ↓ CD68+ and apoptotic cells; (iv) ↓ JNK activation, CRP, TNF and IL-6 transcripts; (v) ↑ mDNA, OXPHOS complexes I and II, ER stress markers; (vi) ↓ GRP78, XBP-1, ATF4, CHOP, and phosphorylated eIF2α; (vii) in vitro: AG and UnAG inhibited steatosis and inflammation with ↑ OXPHOS complexes II, III, and V and downregulated ER stress transducers	A—in vivo; P—in vitro; surgery+ ↑ AG—↓ obesity-associated liver inflammation; ↓ mitochondrial dysfunction, and ↓ ER stress	[87]
	mice; LPS-induced NASH in HFD-fed mice; Kupffer cells and hepatocytes isolated from WT, *GHSR-1a^(−/−)^* or PPARγ*^(+/−)^* mice; eG	(i) ↓ TNF-α and iNOS; (ii) ↑ Arg1 in Kupffer cells treated with LPS	P; GHS-R1a-mediated ↓ of M1 cell polarization; PPARγ mediates the effects of LPS and G on hepatic steatosis	[36]
In vitro	primary rat hepatocytes; eG (Ag and UnAG)	(i) both G isoforms: ↑ intracellular TG content and ↑ mRNA expression of *Mogat2* and *Dgat1*; (ii) AG: ↑ the expression of autophagy-related markers	P; ↑ AMPK-activated mFFA β-oxidation and autophagy	[84]
	normal human liver cells LO2; eG	(i) ↓ lipid accumulation; ↑ autophagosomes in cells; (iii) G-induced lipid clearance associated with ↑ in autophagy; (iv) ↓ mTOR phosphorylation	P; AMPK/mTOR; autophagy	[181]
	primary HSCs (unstimulated and AII-stimulated cells); eG	(i) ↓ expression of collagen-I and TGF-β in HSCs	P; TGF-β	[42]
	HepG2 cells; eG (AG, UnAG)	both isoforms of G: (i) ↓ TNF-α-induced apoptosis and pyroptosis; (ii) AG: ↓ TNF-α-activated hepatocyte autophagy	P; AMPK/mTOR; autophagy	[86]

[↑/↓—increase (activation/stimulation)/decrease (inhibition) level (expression); A—aggravating role; ALI—acute liver injury; AII—angiotensin II; (p)Akt—phospho-Akt (activated protein kinase B); CAT—catalase; CLI—chronic liver injury; AMPK—AMP-activating protein kinase; ATF4—activating transcription factor 4; Bcl-2—B-cell lymphoma 2; BDL—bile duct ligation; C—control; C/EBPα—CAAT/enhancer binding protein-alpha; CCl_4_—carbon tetrachloride; CHOP—CCAAT-enhancer-binding protein homologous protein; COX—cyclooxygenase; DGAT1—diacylglycerol O-acyltransferase-1; ECM—extracellular matrix; (e)G—(exogenous/recombinant) ghrelin; eIF2α—Eucaryotic translation initiation factor 2α; ER—endoplasmic reticulum; (p)ERK—phosphorylated extracellular signal-regulated kinase; eNOS—endothelial nitric oxide synthase; FFA—free fatty acid; GPx—glutathione peroxidase; GRP78—glucose-regulated protein 78; GSH—glutathion; HFD—high-fat diet; HSCs—hepatic stellate cells; HIRI—hepatic ischemia-reperfusion injury; IL-1β—interleukin 1β; IR—insulin resistance; JNK-c—Jun N-terminal kinases; KO—knockout; LC3—microtubule-associated protein light chain 3; LKB1/AMPK—serine/threonine liver kinase B1/AMP—activated protein kinase; LPS—lipopolysaccharide; M1—classically activated macrophages; MDA—malondialdehyde; mTOR—mammalian target of rapamycin; NAFLD—nonalcoholic fatty liver disease; NASH—nonalcoholic steatohepatitis; NF-κB—nuclear factor κB; NO—nitric oxide; (i)NOS—inducible nitric oxide synthase; OS—oxidative stress; OXPHOS—oxidative phosphorylation; P—(hepato)protective role; PGE2—prostaglandin E2; PI3K/Akt—phosphoinositide 3-kinase/Akt pathway; P—protective role; PPARγ—peroxisome proliferator-activated receptor γ; (n-6) PUFA—(omega-6)-polyunsaturated fatty acid; ROS—reactive oxygen species; RYGB—Roux-en-Y gastric bypass; (α)SMA—α smooth muscle actin; SOD—superoxide dismutase; TAA—thioacetamide; TC—total cholesterol; TG(s)—triglycerides; TGF-β1—transforming growth factor beta1; TNF-α—tumor necrosis factor-α; WT—wild-type; XBP-1—X-box-regulated protein 1].

**Table 5:** The Main Immunomodulatory Effects of the Ghrelin System in Colon and Liver Inflammation.

Organ	Local/Systemic Effect	Ghrelin	GHS-R	Obestatin	GOAT
COLON	↓/↑ TNF-α and ↓/↑ IL-1β secretion	X	X	X	
	↓/↑ IL-6 secretion	X			
	↓ IFN-γ secretion			X	
	↑ IL-8 gene expression	X			
	↓/↑ infiltration with neutrophils	X			
	↓/↑ infiltration with macrophages	X	X		
	↑/↓ TLRs		X		
	↑ colitis-induced inflammation				X
	↑ TGF-β and ↑ IL-10	X		X	
	↓/↑ NF-κB	X			
	↑ iNOS and ↑ PGE2 secretion	X			
	↑ COX-2 protein/promoter activity	X	X		
	↑ CAT and ↑ MnSOD activity	X			
	↑ GHS			X	
	↓ MDA	X		X	
	↓/↑ MPO activity	X	X	X	
	↓ proliferation and ↑ apoptosis of Th cells	X			
	↑ GH	X			
	↑ IGF-1	X			
	↓ blood leukocytes			X	
LIVER	↓ TNF-α, IL-1β, and IL-6 secretion	X		X	
	↓ NF-κB	X			
	↓ inflammatory infiltration	X			
	↓ TGF-β and p-Smad3	X			
	↓ MDA plasma/liver	X			
	↓ MPO expression	X			
	↓ NO level	X		X	
	↑ SOD, CAT, GSH, and GPx	X			
	↑ eNOS expression	X			
	↓ COX and PGE2	X			
	↓ iNOS	X			
	↓ apoptosis	X			
	↓ fibrosis	X			

[X—proven impact; CAT—catalase; COX-2—cyclooxygenase-2; GH—growth hormone; GHS-R—ghrelin receptor; GOAT—ghrelin-O-acyltransferase; GPx—glutathione peroxidase; GSH—glutathion; IFN-γ—interferon gamma; IGF-1—insulin-like growth factor 1; IL—interleukin; (e/i)NOS—endothelial/inducible nitric oxide synthase; MDA—malondialdehyde; MnSOD—manganese superoxide dysmutase; MPO—myeloperoxidase; NF-κB—nuclear factor κB; PGE2—prostaglandin E2; TGF-β—transforming growth factor beta; Th cells—T helper cells; TLR—toll-like receptors; TNF-α—tumor necrosis factor-alpha].

## Data Availability

Not applicable.

## Abbreviations

Akt	serine/threonine-protein kinase or protein kinase B (PKB)
APC	adenomatous polyposis coli
BMI	body mass index
cAMP	cyclic adenosine monophosphate
CAT	catalase
CI	confidence interval
CRC	colorectal cancer
CRI	cancer-related inflammation
COX-2	cyclooxygenase-2
DSS	dextran sodium sulphate
eNOS	endothelial nitric oxide synthase
EGFR	epidermal growth factor receptor
ERK	extracellular signal-regulated kinase
GH	growth hormone
GHS-R	ghrelin receptor
GLP-1	glucagon-like peptide-1
GOAT	ghrelin-O-acyl-transferase
GSH	glutathion
HR	hazard ratio
IBD	inflammatory bowel diseases
IGF-1, -2	insulin-like growth factor 1, -2
IR	insulin resistance
JAK	Janus kinase
JNK	c-Jun N-terminal kinases
MAPK	a mitogen-activated protein kinase
MDA	malondialdehyde
MetS	metabolic syndrome
MnSOD	manganese superoxide dysmutase
MPO	myeloperoxidase
mTOR	the mammalian target of rapamycin; protein kinase from PI3K family
NAFLD	nonalcoholic fatty liver diseases
NASH	nonalcoholic steatohepatitis
NF-κB	nuclear factor-kappa B
OR	odds ratio
PGE2	prostaglandin E2
PI3K	phosphoinositide 3-kinase
ROS	reactive oxygen species
SNPs	single nucleotide polymorphisms
STZ	streptozocin
TGF-β	transforming-growth factor beta
TLRs	toll-like receptors
TNBS	trinitrobenzene sulphonic acid
UPR	unfolded protein response
